# Communication Technology Use by Caregivers of Adolescents With Mental Health Issues: Systematic Review

**DOI:** 10.2196/13179

**Published:** 2020-08-19

**Authors:** Ronelle Jansen, Marianne Reid

**Affiliations:** 1 School of Nursing Faculty of Health Science University of the Free State Bloemfontein South Africa

**Keywords:** caregiver, communication technology, adolescent, mental health issues, systematic review, self-efficacy, knowledge, parental skills, IMBP

## Abstract

**Background:**

Caregivers of adolescents with mental health issues experience challenges that may result in the caregivers having a variety of unmet needs. There is a growing need to support these caregivers. Effective support to strengthen positive caregiving behavior in caregivers may address their challenges. Communication technologies offer novel opportunities to assist these caregivers and may contribute to strengthening caregiver behavior. However, little is known about the use of communication technologies among caregivers of adolescents with mental health issues.

**Objective:**

The study aimed to answer the question: “What is the best evidence available to strengthen positive behavior of caregivers of adolescents with mental health issues using communication technology.”

**Methods:**

A systematic review of articles published between January 2007 and August 2018 was conducted. Searches included articles of multiple study designs from EBSCO Host and Scopus platforms with prespecified eligibility criteria. Methodological quality was evaluated using the applicable Critical Appraisal Skills Programme and Joanna Briggs Institute assessment tools.

**Results:**

The search yielded 1746 articles. Altogether, 5 articles met the eligibility criteria and were included in the review for data synthesis. Data analysis and synthesis identified three thematic conclusions reflecting the types of communication technologies used, caregivers as the target population, and strengthening of positive behavior through determinants of the Integrated Model of Behavior Prediction.

**Conclusions:**

The review reported the usefulness of communication technology by caregivers. Caregivers also demonstrated improvement in self-efficacy, knowledge, parent-child communication, and parental skills reflecting positive behavior. Although the use of communication technology is expanding as a supportive intervention to address caregivers’ needs, the evidence for usefulness among caregivers of adolescents with mental health issues is still scarce. More research and information related to preferred methods of communication delivery among caregivers of adolescents is still needed.

## Introduction

### Background

“Nothing about me, without me”

1

The caregiver landscape can be successfully supported through tailored solutions by acknowledging their unique challenges. Caregivers are largely invisible and are mostly underappreciated resources in community health services [2,3]. Caregivers are vital partners who contribute to health care provisioning in communities. They contribute to alleviating resource deficiencies and assist with task sharing in the health sector, despite not formally being part of the health sector [4-6]. Regardless of this important contribution to health care delivery, caregivers receive minimal support from governments [7,8]. Globally, caregivers share a myriad of ongoing challenges and obstacles that are burdensome [2,9], often leading to unmet physical, psychological, and financial needs [10,11].

Caregivers’ own needs are mostly neglected because there is usually more attention focused on the care recipient or the family’s needs [12]. It is thus vital to identify the unmet needs of the caregiver independently from those of the care recipient [9,13]. Unmet needs of caregivers merit more recognition and understanding in order to employ effective support to their prioritized needs [14]. Interventions for caregivers may be beneficial to address needs such as skills training, support, education, and access to resources [15,16].

Fortunately, there is greater awareness of mediating interventions to support caregivers who are providing care for relatives with a chronic illness or disability [17]. Various interventions have been geared toward caregivers of persons presenting with dementia [14,18,19], cancer [20,21], HIV [22,23], chronic diseases [24-26], maternal and child health [27,28], and long-term conditions in children [29,30]. Various authors [31,32] claim that there is limited research available that focuses on families caring for adolescents with mental health needs, while Cardamone-Breen et al [33] stated there is a scarcity of parental interventions to prevent them from internalizing the mental health issues of adolescents. Parent- and family-focused interventions for child and adolescent mental health care are poorly represented in research [34-36]. Authors do not use similar terminology when referring to mental health issues, problems, illnesses, or conditions. This review will refer to *mental health issues* as a broad term for mental, emotional, and behavioral problems or disorders focusing on depression, anxiety, and substance use. Depression, anxiety, and substance use disorders are more common among adolescents [37-39]

Armoiry et al [40] reported equivocal findings regarding the use of communication technologies among families of young people with long-term conditions. Rodríquez-Meirinhos et al [31] also highlighted caregivers’ need for education and information in preparation for caring for an adolescent with a mental illness. These authors [31] also established the necessity for dependable interventions to support families and community services to empower this group of caregivers.

Evidence-based supportive interventions that are flexible and developed according to the needs of caregivers may reduce caregiver burden and improve mental health caregiving [9]. Kuhn and Laird [41] stated that a combination of supportive interventions would be more useful for a diverse population, allowing tailored communication. Understanding caregivers’ cultural context is important to develop a tailored intervention congruent to their specific needs [6,9,42]. Moreover, caregiver interventions should be sustainable [43], easily accessible [44], available [9], culturally, ethnically, and linguistically tailored [45], and caregiver-focused [46].

Literature suggests that communication technologies are arising as an acceptable intervention to assist caregivers of children and young people in the management of conditions [40]. Communication technologies terminology is labeled interchangeably in the literature but refers to computers, the internet, electronic health (eHealth), and mobile health (mHealth), including networks and media services to transmit information [47-50]. This taxonomy of communication technologies is often used simultaneously in the research of interventions reporting on various outcomes [51]. Combined communication technology interventions often result in effective health outcomes and are feasible substitutes for traditional health promotion approaches such as printed material [52,53]. Vergunst [54] also recommended that creative interventions need to be established for caregivers in resource-poor settings such as rural areas to bridge gaps in mental health service delivery for the individual, the family, and the community. Although communication technology interventions are potentially valuable to support caregivers of children with mental and behavioral problems [55], limited data are available even though it is a major public health concern [56]. The emergence of communication technologies to deliver training for caregivers is increasing and show promise in real-world setting [57].

Evidently, the usefulness of communication technology interventions for caregivers of adolescents with mental health issues differs. DeHoff et al [58] found that parents of children with special needs got support through an online social platform to accept and manage the child’s condition. Another study disclosed that parents of youth with mental health problems seem to be positive about the use of computer-based therapies [59]. A web-based health promotion program utilized by adolescent girls and their mothers for drug use, among other conditions, resulted in positive health behavior changes [60]. The study by Russell et al [61] indicated a high satisfaction among parents of children with behavior problems when engaging in a computer-based education program.

The novelty of communication technologies in the management of psychiatric and mental health illnesses may be valuable to investigate [62]. According to Casale et al [63] and Robila [64], there is a need to support parents through interventions that provide resources to improve parenting, particularly in reducing adolescent behavioral and emotional problems. It would appear that communication technologies are beneficial in supporting caregivers in improving their skills, abilities, self-efficacy, and knowledge [65,66]. A review of caregivers caring for adolescents with developmental disabilities similarly highlighted the effect of tailored interventions to augment caregivers’ self-efficacy, self-esteem, positive coping skills, and supportive social networks [67].

### Theoretical Framework

The Integrative Model of Behavior Prediction (IMBP) endorses determinants such as attitudes, norms, and self-efficacy, by predicting behavior. Consequently, these determinants are based on underlying beliefs that, in turn, influence the intention to perform a specific behavior. Behavior may be challenged due to a lack of skills or environmental constraints [68,69].

The IMBP guided this review in clarifying caregivers’ behavioral intention for utilizing communication technologies by determining their belief, attitude, norms, self-efficacy, skills, environment, and intention. Behavioral intention can be predicted if the populations’ beliefs, attitudes, norms, self-efficacy, skills, and environment are known [68,70,71]. To our knowledge, however, there have been no systematic efforts to synthesize evidence on the usefulness of communication technologies to strengthen positive behavior in caregivers of adolescents with mental health issues. Any positive response to a determinant was regarded as strengthening [71,72] of caregivers’ behavior toward the adolescent.

The authors conducted a systematic review to collect and synthesize all evidence fitting the prespecified eligibility criteria. The authors sought to answer the following question based on the PICO format [73] (see Table 1 for the application of the PICO format): “What is the best evidence available to strengthen positive behavior of caregivers of adolescents with mental health issues using communication technology?” This paper provides a critical review and synthesis of the best available evidence about the use of communication technologies by caregivers of adolescents with mental health issues.

**Table 1:** Application of the PICO format.

PICO	Application
Population	Caregivers of adolescents with mental health issues (Caregivers namely families, parents, sibling, carer, etc)
Intervention	Communication technology
Comparison	Routine communication technology
Outcome	IMBP^a^ determinants (beliefs, attitude, norms, self-efficacy, skills, environment, and intention)

^a^IMBP: Integrated Model of Behavior Prediction.

## Methods

### Design

The authors undertook a systematic review that included multiple study designs [73-75]. This systematic review has been registered in PROSPERO (CRD42018094680) and was conducted according to PRISMA guidelines.

### Search Strategy

A senior research librarian conducted a systematic search on electronic databases that covered articles published from January 1, 2007 to August 2, 2018. Databases from the EBSCO Host platform (PsycINFO, Academic Search Ultimate, MEDLINE with Full Text, Health Source: Nursing/Academic Edition, SocINDEX with Full Text, CINAHL with Full Text, ERIC, CAB Abstracts, MasterFILE Premier, Africa-Wide Information, PsycARTICLES, OpenDissertations, Communication & Mass Media Complete, Business Source Ultimate, SPORTDiscus with Full Text, Health Source - Consumer Edition, Humanities Source, EconLit with Full Text, GreenFILE) and the Scopus database were included. No language or study design restrictions were applied. Search strings determined by the authors in collaboration with the research librarian were used to retrieve articles from the abovementioned databases. It was decided to make use of free text, as the search would be conducted on multiple databases, of which only MEDLINE and CINAHL utilize controlled vocabulary (MeSH and CINAHL subject headings respectively). Before conducting the search, a concept analysis was done to identify synonyms to be used in the free-text search (see Table 2 for a combination of the search string using Boolean operators). The first author (RJ) also searched the reference lists of retrieved articles. Dissertations and book chapters formed part of the retrieved articles.

**Table 2:** Search strings used.

Search strings
caregiver* OR “care giver*” OR family* OR families* OR parent* OR mother* OR father* OR sibling* OR carer OR carers OR “lay worker*” OR “next of kin” AND
“mental* health*” OR “mental* ill*” OR “mental* disorder*” OR “mental* diseas*” OR depress* OR anxiet* OR substance AND
teen* OR adoles* OR juvenile* OR youth* AND
belief* OR conviction* OR faith OR trust* OR norm OR norms OR custom* OR attitude* OR outlook* OR approach* OR Self-efficac* OR ability* OR Skill OR skills OR expertis* OR able OR abilit* OR talent* OR proficien* OR knowhow OR capabilit* OR knack OR competen* OR Intent* OR determination* OR planning OR resolve OR decide* OR decision* OR choose OR select* OR choice* AND
mobile* OR cell* OR smart OR sms OR “short message service*” OR text* OR device* OR mhealth* OR m-health* OR ehealth* OR e-health* OR “instant messag*” OR app OR apps OR phone* OR smartphone* OR “electronic device*” OR “portable device*” OR “phone intervention*” OR “telephon* intervention*” OR online*) not (“stem cell*” OR “sickle cell*” OR ”assist“ device*” AND
mobile* OR cell* OR smart OR sms OR “short message service*” OR text* OR device* OR mhealth* OR m-health* OR ehealth* OR e-health* OR “instant messag*” OR app OR apps OR phone* OR smartphone* OR “electronic device*” OR “portable device*” OR “phone intervention*” OR “telephon* intervention*” OR online* OR caregiver* OR “care giver*” OR family* OR families* OR parent* OR mother* OR father* OR sibling* OR carer OR carers OR “lay worker*”

### Eligibility Criteria

Articles were eligible for inclusion if they (1) focused on caregivers such as families, parents, siblings, or relatives of adolescents with mental health issues (concentrating on depression, anxiety, and substance use disorders) as the target population, (2) reported on communication technology usage, and (3) described caregiver determinants according to the IMBP. Articles were excluded if they did not meet the inclusion criteria or (1) if they focused only on adolescents as the target population, (2) if they reported on non–communication technology interventions, and (3) if the focus was on mental health–related therapies or traditional communication interventions.

### Selection Procedure

The first author (RJ) filtered all titles and abstracts obtained from the search against the review question and eligibility criteria. The second author (MR) verified articles for compliance with eligibility criteria. A large number of articles were excluded, and duplicates were removed. Full-texts for the remaining articles were obtained from the librarian. These full-text articles were independently screened for eligibility by both authors, and any discordances were resolved through discussion. Additionally, references of selected articles were screened further for relevant studies.

### Quality Appraisal

The first author (RJ), together with two senior researchers, rigorously evaluated the quality of the selected articles according to the Critical Appraisal Skills Programme [76] and Joanna Briggs Institute assessment tools applicable to each study design [77]. All tools evaluated the appropriateness of methods used, clarity of focus, the recruitment process, the accuracy of measures used, data collection, presentation and analysis, clarity in the statement of the findings, and appropriateness of context. If more than two aspects were not addressed in the articles, it was excluded. This was depicted as either Level 1* (full marks obtained) or Level 1 (–1 mark) in Table 3. Table 3 includes a hierarchy classification system in terms of research design, according to the American Dietetic Association [78,79]. This critical appraisal of the full-text articles determined the selection and inclusion of articles in this systematic review.

**Table 3:** Summary of included articles’ study designs.

Extracted data		Reference				
		Deitz et al [80]	Cardamone-Breen et al [33]	Choi et al [81]	Molleda et al [82]	Estrada et al [83]
Design		Randomized control trial	Randomized control trial	Quasi-experimental	Qualitative, descriptive	Qualitative interviews
**Grading**						
	Level	1	1	1*	1	1
	Hierarchy	A	A	C	D	D
**Communication technology**						
	Type	Web-based	Web-based	Web-based	Internet-based, eHealth	Internet-based, eHealth, tablets
	Reminders	Multiple—not specified	—	Phone calls, text messages, and emails	—	Phone or text messages
	Financial support	National Institute on Mental Health	National Health and Medical Research Council	Korean government	—	Centers for Disease Control and Prevention (grant)
	Recruitment	—	Online networks, social media, computer, online parent portals, and email communication	Via online communities	Emails	Online
	Follow-up		Phone calls, text messages, and emails			
**Targeted**						
	Caregivers	Parents, n=99	Parent/caregiver, n=349	Parents, n=114	Parents, n=6	Parents, n=29
	Youth/ adolescents	Mental health problems or issues	With depression and anxiety disorders	Mental health problems or issues (common mental health problems (ie, bullying, depression, internet addiction, and suicide)	With drug use and risky sex behavior	With drug use and sexually risky behavior
Setting		Workplace of parents: ISA Group in Alexandria in the US	Secondary schools in Australia	Two elementary schools and four middle schools from four cities in Korea	Pediatric mobile clinic in Miami-Dade County and another clinic located on the university medical campus (University of Miami Miller School of Medicine)	Multiple middle schools relatively close to where the families lived and at the University of Miami offices.

### Data Extraction, Analysis, and Synthesis

The authors extracted specific data from the selected articles in a tabular format to record the study characteristics, types, and delivery method of communication technologies and the strengthening of positive behavior according to IMBP determinants. A meta-analysis was not feasible due to the heterogeneity of the included articles. A thematic data analysis [84-86] took place and both authors discussed the themes to refine the data according to the review question. Subsequently, the data were organized into relevant thematic conclusions addressing the research question based on the PICO elements. After that, the results were synthesized across all articles.

## Results

### Search Results and Selection of Articles

**Figure 1:**
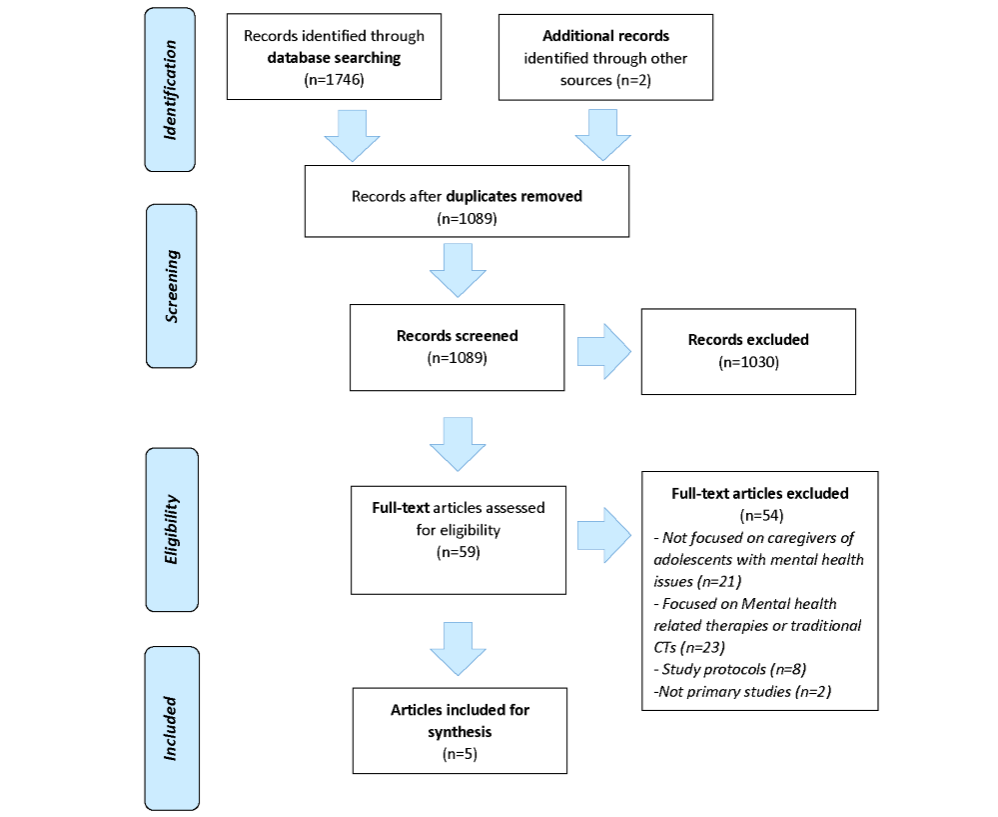
PRISMA flow diagram.

The search identified 1746 electronic records of possible interest. Two additional articles were added, one through contacting an author and another through reference list checking. After electronic and manual removal of duplicates, 1089 records remained. Further screening for eligibility of identified records resulted in the retrieval of 59 full-text articles that were potentially relevant for analysis. The authors then conducted a comprehensive review of these full-text articles and included articles that specifically focused on communication technologies used by caregivers of adolescents with mental health issues. A total of 5 articles met all the eligibility criteria. Figure 1 presents the PRISMA flow diagram of the study, and Table 3 presents a summary of the included articles’ study design evaluations.

### Characteristics of Included Articles

The final articles included in this systematic review consisted of multiple designs. There were 2 randomized controlled trials [33,80], 1 quasi-experimental study [81], and 2 qualitative studies [82,83]. In all 5 articles, the caregivers had been exposed to communication technologies in caring for adolescents with mental health issues. Of the 5 studies, 3 studies were performed in the United States [80,82,83], 1 was performed in Australia [33], and 1 was performed in Korea [81]. Articles varied in methodology, intervention, outcomes, measurement scales, and findings. Not all of the articles complied with all of the identified IMBP determinants leading to positive behavior. Table 4 presents a summary of the results reported by the 5 articles.

Results from the 5 articles were synthesized and thematically analyzed to formulate conclusions. The thematic conclusions are discussed according to the types of communication technologies used, caregivers as the target population, and strengthening of positive behavior through IMBP determinants.

**Table 4:** Summary of results reported by the 5 articles.

Reference	Strengthening positive behavior according to determinants within IMBP^a^
Deitz et al [80]	Total knowledge score: significantly greater for the experimental group than the control group (*t*=–7.96, *P*<.001) Knowledge of treatment: statistically significant (*t*=–2.92, *P*=.006) A significant difference was found between experimental and control groups on self-efficacy in handling mental health issues in their children (*F*=12.73, *P*=.001). Significant paired *t* test analysis of increases in self-efficacy from pretest to posttest among the individuals receiving the intervention was found (*t*=–3.20, *P*=.003). No significant differences between groups on the measures of family communication, adaptability, cohesion, and attitudes toward mental health issues were found.
Cardamone-Breen et al [33]	Chi-square analyses of the postintervention (1-month) assessment revealed a significant difference in attempts to change parenting (n=307, χ_3_^2^=19.65, *P*<.001), with significantly more intervention group parents reporting making some changes to their parenting. The majority of parents (93.6%) reported they were somewhat or very satisfied with the feedback received, and 95.1% reported the feedback as either somewhat, very, or extremely useful. Most parents (90.2%) reported they were somewhat or very likely to change their parenting based on the feedback provided. Conclusion: accessible, low-cost preventive approach
Choi et al [81]	Participants in the intervention, compared to those in the control group, demonstrated preliminary evidence of improved parental knowledge. The effect size of parental knowledge was large (d=0.60; 95% CI 0.21-0.99). Parents in the intervention group showed increased parental self-efficacy, parent-child communication, and satisfaction with parent-child relationships, and decreased parent-child conflict. Parents reported accessibility and convenience to complete the intervention.
Molleda et al [82]	Effective parent-adolescent communication skills. Parents’ positive experience with flexibility, accessibility, and convenience in delivery of eHealth. Parents also reported the ability to apply the lessons learned from eHealth *Familias Unidas* to their daily lives.
Estrada et al [83]	It was feasible to recruit, engage, and retain Hispanic families into an eHealth intervention and deliver it electronically. Positive feedback was provided by the parents regarding eHealth. Parents stated there were multiple lessons learned from engaging in eHealth *Familias Unidas*: effective parent-adolescent communication and active parental attention and involvement in an adolescent’s life. Culturally appropriate online content allowed parents to access sessions at their convenience and minimized costs for researchers and participants alike

^a^IMBP: Integrated Model of Behavior Prediction.

### Types of Communication Technologies Used

All the studies implemented web-based interventions [33,80-83]. Content employed via the communication technologies and delivery thereof varied considerably. The web-based program by Deitz et al [80] consisted of 4 multimedia modules that provided information regarding symptoms and treatment options for depression and anxiety, building parental skills, and additional resources with information about mental health issues. Parental skills included communication, relationships, and healthy lifestyles. Estrada et al [83] and Molleda et al [82] adapted an evidence-based intervention, *Familias Unidas*, into a web-based intervention consisting of 8 e-parent group sessions of video recordings and 4 online family sessions (parent-adolescent) with a facilitator. Choi et al [81] reported on a 4-week web-based intervention (Stepping-stone) containing educational sessions, media files, verbal feedback, weekly assignments, and practice sessions regarding bullying, depression, suicide, and communication skills. Cardamone-Breen et al [33] adapted a single-session web-based component of the Partners in Parenting intervention. This intervention provided personalized feedback and psychoeducation concerning guidelines on adolescent depression and anxiety after the parents completed an online survey to assess parenting practices.

### Target Population

The study populations in the included articles predominantly comprised caregivers as parents or family members. Deitz et al [80] used parents or caregivers of youth between the ages 5 and 21, while Molleda et al [82] included clinic personnel, facilitators (ie, physicians, nurse practitioners, administrators, and mental health workers), and parents or primary caregivers of Hispanic adolescents (age range: 12 to 16 years) [82]. Estrada et al [83] also included Hispanic families with parents and adolescents between the ages of 12 and 16 [83]. The study by Choi et al [81] encompassed parents who had at least one child aged 11 to 16 years. Cardamone-Breen et al [33] used a community sample of parents, together with adolescents aged 12 to 15 years old. The most common problems among adolescents were mental health or behavioral issues [80,81], depression and anxiety disorders [33,81], bullying [81], suicide [81], and substance use [82,83]. The majority of participants in the studies were female, recruited from various urban settings, and educated.

### IMBP and Other Determinants

Outcomes focusing on the IMBP determinants in the current review were about self-efficacy and skills such as parent-child communication: 2 studies [80,81] measured the effect of the intervention on caregiver self-efficacy that improved after the intervention; 3 studies [81-83] also demonstrated the effectiveness of communication skills between caregiver and adolescent, and 1 randomized controlled trial [80] measured no significant differences between study groups in family communication skills. Of the 5 studies, Cardamone-Breen et al’s study [33] showed promising changes in parenting skills and behavior, and another study [80], a randomized control trial, reported outcomes regarding parental attitudes demonstrating no significant differences between the two study groups.

Another observation was that effective parental skills resulted in satisfactory parent-child relationships as well as a decline in parent-child conflict [81]. Caregivers similarly appreciated the feedback they received regarding their parental skills after using the intervention [33] and lessons learned to implement in their daily lives [82,83].

We identified 2 studies [80,81] that measured the effect of communication technologies use on caregivers’ knowledge showing an improvement. Additionally, Estrada et al [83] found that parents were more involved and attentive in the life of adolescents after engaging with the eHealth intervention. In turn, participants in Estrada et al’s study [83] gave positive feedback regarding the use of the eHealth intervention while Molleda et al [82] found that parents experienced the eHealth delivery as positive.

## Discussion

### Principal Findings

The review results show the potential value of communication technologies to strengthen caregivers’ behavior when caring for an adolescent with mental health issues. Overall, the results yielded evidence of the usefulness of communication technologies by caregivers. Moreover, the results contributed to the limited literature on communication technology interventions for caregivers caring for the adolescent with mental health issues.

As shown by this study, the results were mostly positive, which may raise questions about selection bias. Aligned to the review question, we were interested in determinants showing improved outcomes among caregivers’ when using communication technologies. It becomes particularly imperative to recognize users’ preferences and acceptance of communication technologies in a health care setting. Identifying appropriate communication technologies to inform and support caregivers may be a challenge, but it is essential to understand their adoption thereof [87,88]. Information dissemination delivered through communication technologies is gradually replacing traditional approaches [89], but the implementation in health care [90] and among caregivers [14,91] is still lacking.

Communication technologies can distribute an unprecedented amount of information through technology such as electronic devices, system software, and information networks such as the internet, which provides access to resources [92,93]. Moreover, Schneider’s [94] systematic review reported that utilizing communication technologies to gather and stream information received the highest proportion of use. Multimedia approaches including internet and web-based programs are a means for providing informational support to caregivers; they also found that effective parent education through communication technologies can augment the mental well-being of children [95]. Our results are consistent with earlier reports in this field that suggest that web-based interventions are effective for caregivers managing mental health issues in adolescents [91,96,97].

A prevailing expectation of communication technologies is that it might improve knowledge acquisition regarding illnesses. However, applicable information should be according to the caregivers’ specific needs [98]. Statistically significant differences were found related to parental outcomes of knowledge, attitudes, and skills in efficient web-based interventions [99]. Exploiting the internet for knowledge about chronic diseases, as noted by Mahmud et al [53], can assist individuals and communities in health promotion. In contrast, low-income parents were not confident in using the internet and could not distinguish between good- or bad-quality information [100]. Sweeney et al [59] concluded that parents of youth with mental health problems demonstrated poor knowledge regarding computer-based therapies but were positive about using it.

Communication technologies are now mature enough to enable learning and knowledge exchange among caregivers in health care [101]. This functionality is attributable to the fact that communication technologies are associated with boosting the health care landscape through information exchange and transformation among large populations [102]. Our review indicated some evidence that computer-based interventions among large groups of parents are less expensive. The financial implication is particularly relevant in the current global socioeconomic climate, especially in low-resource settings that should keep cost-effectiveness of communication technologies in mind [103] when serving vulnerable populations such as caregivers in deploying health care [104]. According to Sprague et al [105], the world still experiences barriers to internet adoption despite high technology penetration because of disproportionately rural populations, low-income, illiteracy, elderly users, and female users. Therefore, it is vital to explore the best evidence available related to communication technologies barriers such as delivery methods, cost-effectiveness, caregiver characteristics, and outcomes, which will predict adoption thereof.

None of the included studies measured text messaging, phone calls, mobile apps, or social networking as a favored network delivery; however, included studies mentioned the use of communication technologies to recruit participants and send reminders. Domek et al [106] and Anderson-Lewis et al [107] suggest that text message interventions may be useful in rural families and have the potential to disseminate public health information. Mobile apps show some promise in serving families of youth with mental health issues in resource-constraint settings [108]. Breitenstein et al [109] also determined that digital delivery, such as mobile apps, might theoretically be cost-effective, sustainable, and reach large numbers for parent training. Furthermore, some studies [110,111] have shown that social media effectively supports and informs caregivers through shared participation. Catalano et al [112] identified that parental social support and interacting improved caregiver well-being.

Collective participation is valuable, but researchers should be mindful of caregivers’ individuality when exploring the usage of communication technologies. It is important to conceptualize the characteristics of caregivers in the context of communication technologies acceptance [113-115]. Caregivers of adolescents are typically a parent or family member who assumes a central role in caregiving. Transition into the caregiver role can be different for each caregiver. Recognizing the individual needs of each caregiver is crucial when investigating the usability of communication technology interventions [9,116], especially in caregivers of children or adolescents with mental health issues [67,117,118].

Caregivers’ adoption of tailored web-based interventions focusing on their needs may improve their resourcefulness and mobilize effective caregiving [119,120]. Tailored communication technologies was highlighted in this review, indicating contextual relevance related to the usage of communication technologies. Besides, this review also highlighted common elements depicting their approval of utilizing communication technologies, such as the accessibility, cost-effectiveness, convenience, and flexibility thereof. Generally, study participants gave positive feedback related to communication technologies use. Modifying communication technology interventions to match caregivers’ preferences might lead to favorable changes in parenting practices and satisfaction in intervention utility [121]. Overall, the findings demonstrated the acceptability of communication technologies by caregivers, and the use of communication technologies was associated with improved caregiver and mental health outcomes among adolescents.

Some studies in this review identified diverse interventions that facilitated the strengthening of caregivers’ behavior, such as improved self-efficacy, enhanced knowledge, and better parent-child communication skills and practices. These findings complement those of other studies in that online tools have proved to be successful in improving caregivers’ knowledge, skills, coping [122,123], and self-efficacy [120,124]. Nieuwboer et al [99] summarized that the internet supplies information, support and advice to parents with different needs that encourage changes in their parental abilities. Furthermore, technology provides access to others for building support and knowledge through positive engagement [125]. Reportedly, the potential impact of communication technologies on caregivers’ well-being is ubiquitous and may guide their behavior toward the care recipient [126].

Caregivers who feel that they can perform behavior effectively will continue to repeat that specific behavior. This performance is based on knowledge, skills, and self-efficacy. A repetition of the behavior will occur if it is associated with a positive feeling and a sense of confidence; the accomplishment of the behavior, reflects self-efficacy and competency in performing the task [127,128]. People hold specific beliefs about behavior and intentionally perform according to that belief. From the IMBP, people who have the necessary skills will perform more satisfactorily, which will lead to favorable outcomes [129]. Hall and Bierman [130] reported improvement in parental knowledge and attitude through technology-based interventions. Of course, targeted interventions strengthening parental knowledge, self-efficacy, and skills may lead to improved child mental health outcomes [100] and positive parental outcomes [9].

Thus, it is legitimate to say that a communication technologies–based intervention has great potential to be used by caregivers to reinforce positive behavior when caring for an adolescent with mental health issues. Communication technologies also represent possibilities to provide support to caregivers by addressing their individual needs which could be highly convoluted. This review shows a need for further research in the area of supporting caregivers of adolescents with mental health issues.

### Strengths

The authors conducted a comprehensive review following a stepwise methodological process for a systematic review. We included multiple study designs to understand how communication technologies may strengthen caregivers’ behavior and improve caregiver outcomes. We used standardized checklists to appraise selected articles critically. The results provided knowledge related to the use of communication technologies among caregivers of adolescents with mental health issues.

### Limitations

We might have missed potentially relevant articles, although explicit inclusion and exclusion criteria were set up. The first author initially screened titles and abstracts independently for applicability while some were verified by the second author for quality control purposes. This review was limited to articles describing caregiver outcomes according to the IMBP determinants, and this restriction may have eliminated meaningful information from other communication technology advantages.

### Conclusions

The review results indicate that using communication technology is useful to strengthen caregivers’ behavior by providing information and resources. Additionally, a better understanding of caregivers’ attitudes and environmental constraints toward communication technologies may inform optimal usefulness thereof. Evidence for caregivers of adolescents with mental health issues using communication technologies is not readily available in the literature. There was limited empirical research outlining the methods of communication delivery, and it is worth exploring this in future research. Besides, future research should focus on the development of more innovative communication technology interventions for caregivers in different contexts and scaling up of efforts to implement them for improved mental health care among adolescents.

## Abbreviations

eHealth: electronic health
IMBP: Integrated Model of Behavior Prediction
mHealth: mobile health
PICO: population, intervention, comparison intervention, outcome
